# The Eurolight project: the impact of primary headache disorders in Europe. Description of methods

**DOI:** 10.1007/s10194-011-0356-y

**Published:** 2011-06-10

**Authors:** C. Andrée, L. J. Stovner, T. J. Steiner, J. Barré, Z. Katsarava, J. M. Lainez, M. Lanteri-Minet, G. Mick, D. Rastenyte, E. Ruiz de la Torre, C. Tassorelli, P. Vriezen, C. Lampl

**Affiliations:** 1 Center of Public Health Research (CRP-Santé), 1A, rue Thomas Edison, 1445 Strassen, Luxembourg; 2 Department of Pharmaceutical Sciences, University of Basle, Basle, Switzerland; 3 Norwegian National Headache Centre, St. Olavs University Hospital, Trondheim, Norway; 4 Department of Neuroscience, Norwegian University of Science and Technology, Trondheim, Norway; 5 Department of Neuroscience, Imperial College London, London, UK; 6 Department of Neurology, University of Essen, Essen, Germany; 7 Department of Neurology, Hospital Clinico Universitario, University of Valencia, Valencia, Spain; 8 Centre Hospitalo-Universitaire de Nice, Nice, France; 9 Pain and Headache Unit, Regional Pain Network, Voiron, France; 10 Lithuanian University of Health Sciences, Kaunas, Lithuania; 11 Asociacion Española de Pacientes con Cefalea, Valencia, Spain; 12 Centro Italiano di Ricerche Neurologiche Applicate (CIRNA) and Headache Science Centre, National Neurological Research Institute C. Mondino Foundation, University of Pavia, Pavia, Italy; 13 Nederlandse Vereniging van Hoofdpijnpatiënten, Amersfoort, Netherlands; 14 Department of Neurology and Pain Medicine, Konventhospital Barmherzige Brüder, Linz, Austria

**Keywords:** Eurolight, Primary headache, Methodology, Impact, Burden

## Abstract

The Eurolight project is the first at European Union level to assess the impact of headache disorders, and also the first of its scale performed by collaboration between professional and lay organizations and individuals. Here are reported the methods developed for it. The project took the form of surveys, by structured questionnaire, conducted in ten countries of Europe which together represented 60% of the adult population of the European Union. In Lithuania, the survey was population-based. Elsewhere, truly population-based studies were impractical for reasons of cost, and various compromises were developed. Closest to being population-based were the surveys in Germany, Luxembourg, the Netherlands, Italy and Spain. In Austria, France and UK, samples were taken from health-care settings. In addition in the Netherlands, Spain and Ireland, samples were drawn from members of national headache patient organizations and their relatives. Independent double data-entry was performed prior to analysis. Returned questionnaires from 9,269 respondents showed a moderate female bias (58%); of respondents from patients’ organizations (*n* = 992), 61% were female. Mean age of all respondents was 44 years; samples from patients’ organizations were slightly older (mean 47 years). The different sampling methods worked with differing degrees of effectiveness, as evidenced by the responder-rates, which varied from 10.8 to 90.7%. In the more population-based surveys, responder-rates varied from 11.3 to 58.8%. We conclude that the methodology, although with differences born of necessity in the ten countries, was sound overall, and will provide robust data on the public ill-health that results from headache in Europe.

## Introduction

Primary headache disorders in Europe, as elsewhere in the world, are common, disabling and costly [1–3]. They are also under-recognized and undertreated, so that the burden of headache in Europe remains unnecessarily high [1]. Amongst the several reasons contributing to this is a lack of political awareness of the scope and scale of the burden of headache, which itself is attributable to incomplete knowledge of these.

The Eurolight project, a collaborative data-collection exercise in ten countries of Europe initiated by the Center of Public Health Research (CRP-Santé) Luxembourg and supported by the European Agency for Health and Consumers (EAHC), was designed to address this knowledge gap. More specifically its purposes were to estimate, in Europe, the prevalence and impact of headache disorders of public-health importance—migraine, tension-type headache (TTH) and chronic headache disorders occurring on ≥15 days/month—and use this information to raise awareness, among the general public, health-care professionals, health policy-makers and governments of Europe, of headache as a major cause of public ill-health. Ultimately, its aim was to contribute to the improvement of health care for headache and the quality of life of people affected by headache disorders.

This paper describes the structure, organization and methods of the project.

## Project description

The project took the form of surveys by structured questionnaire, conducted from November 2008 to August 2009, of population samples from ten countries of Europe representing 60% of the adult population of the European Union: Austria, France, Germany, Ireland, Italy, Lithuania, Luxembourg, Netherlands, Spain and UK.

The basic methods were developed, tested in a pilot study in Luxembourg [4] and then refined and finalized, with differences in each country (described below).

## Partners and committees

The Eurolight project brought together 25 partners from 15 countries: 2 public bodies (CRP Santé, Luxembourg, and Regione Lombardia—Sanità, Italy); clinicians from 11 hospitals; the professional European Headache Federation (EHF); 9 European patients’ organizations including the European Headache Alliance (EHA); the World Headache Alliance (WHA); and *Lifting The Burden* (LTB), a non-governmental organization directing the Global Campaign against Headache under the auspices of the World Health Organization (WHO). The project was directed by a Project Steering Committee (PSC) and overseen by an External Evaluation Board (EEB).

The clinician partners and headache expert members of the PSC were responsible for scientific quality; the patients’ organization partners ensured relevance of the project to people affected by headache; LTB contributed methodological expertise acquired in the public health context. The roles of the PSC were to develop the protocol according to scientific principles, review progress, circumvent practical difficulties, ensure quality control, review the data, plan the analysis and formulate recommendations for future action contingent upon the findings. The EEB was responsible for external quality evaluation of the project with respect to scientific relevance (new knowledge, evidence base and validated content), patient relevance (all relevant contributors to impact of headache), ethical aspects (ensuring ethics approvals where needed) and dissemination. At least one EEB member was invited to all PSC meetings.

## Ethics

The National Ethics Committee of Luxembourg gave overall approval of the protocol. Further approvals were obtained from national or local ethics committees wherever needed as the methods for recruitment of participants differed between countries.

Similarly, data protection approvals were obtained centrally in Luxembourg and at country levels in compliance with national and European privacy laws.

All potential participants in the project were informed about the purpose and nature of the study. In most countries, where questionnaires were mailed, printed information leaflets in the language of the expected recipient were enclosed with them. In the Netherlands internet survey, this information was provided online. In the Lithuania door-to-door survey, leaflets were handed directly to prospective interviewees and this information was supplemented verbally as required.

## Questionnaire development, validation and translation

The Eurolight questionnaire was based on the BURMIG questionnaire, which itself was developed for the BURMIG (burden of migraine) study, a Eurolight pilot study in Luxembourg [4]. Modifications were made in the light of the results of that study, and some elements were imported from other validated sources. Full details of the development, content and validation of the Eurolight questionnaire have been described previously [5].

Initially drafted in English, it was first tested among lay people for intelligibility and face validity, revised as necessary and then translated into Dutch, French, German, Italian, Lithuanian, Luxembourgish, Portuguese (for part of the population in Luxembourg) and Spanish in accordance with the LTB translation protocol for hybrid documents [6]. The translated versions were tested for comprehensibility, internal consistency and test–retest reliability in 426 headache patients in Austria, France, Germany, Italy, Spain and UK.

## Diagnosis of headache and assessment of impact

Demographic, screening (for headache) and headache-diagnostic questions (the last based on the international classification of headache disorders, 2nd edition (ICHD-II) [7]) were supplemented by several question sets addressing impact, together totalling 103 items.

Only one headache type was diagnosed in each respondent, those reporting headaches of more than one type were asked to focus on the one most bothersome to them. The diagnostic questions were imported, with linguistic adaptation by the PSC as necessary, from the epidemiological questionnaire developed by LTB and used in India (unpublished), China [8] and Russia [9] to differentiate migraine from TTH and identify probable medication-overuse headache (MOH) amongst other headaches occurring on ≥15 days/month. Diagnoses in respondents with headache on <15 days/month (episodic headache) were derived, from the responses to these questions, by means of a computerized algorithm constructed by LTB for this question set and applying ICHD-II criteria [7]) for, in order, migraine, TTH, probable migraine and probable TTH.

Further questions enquired into frequency, intensity and duration of headache, use of health-care resources (medication, consultations, investigations and hospitalizations) and effects of headache on school, work, career, income, family life, children and household partner. In addition there were standard questionnaires on lost time (HALT index [10]), quality of life (WHOQoL-8 [11]) and anxiety and depression (HADS [12]).

## Study populations and sampling methods

The countries participating in the survey (see below) were, mainly, those of the members of the PSC. They were selected as a diverse mix of European countries in terms of population size, health-care system and level of income.

In Lithuania, a country of the former USSR, a region for which little prior knowledge existed of the prevalence of headache, a sample was derived from the general population. In the other countries, all in Western Europe, true population-based studies were impractical for reasons of cost, and various compromises were developed. Closest to being population-based were the surveys performed in Germany, Luxembourg, the Netherlands, Italy and Spain. In Austria, France and UK, samples were taken from health-care settings. In addition in the Netherlands, Spain and Ireland, samples were drawn from members of national headache patient organizations and their relatives.

Full details of sampling methods in each country are set out below. A summary of the data collection methodology and the sampled populations is given in Table 1.

**Table 1:** Summarized methodological description of the surveys in ten countries

Survey	Target population and mode of distribution of questionnaire
Studies with a general-population basis or conducted in health-care settings	
Austria	Consecutive patients consulting GPs or neurologists for any reason; questionnaire handed directly
France	Consecutive patients consulting GPs for any reason; questionnaire handed directly
Germany	Random general-population sample from urban and rural areas, contacted by regular post
Italy	Stratified general-population sample from urban and rural areas, contacted by regular post
Lithuania	General-population sample in and around Kaunas (urban and rural), contacted by door-to-door cold-calling and personally interviewed by trained medical students
Luxembourg	Stratified general-population sample contacted by regular post
Netherlands-population	Stratified general-population sample contacted by internet
Spain-workplace	Stratified sample of postal services employees, contacted by internal post by occupational health physicians
UK	Consecutive patients attending GPs for any reason; questionnaire handed directly
Studies among members of headache patients’ organization	
Ireland	Members of MAI and their non-biological relatives, contacted by regular post
Netherlands-patient	Random sample of members of NVvHP and (where existing) their non-headache-affected partners, contacted by regular post
Spain-patient	Members of AEPAC and their family; questionnaire distributed by hand via helpers of AEPAC
Studies among non-responders	
Germany-nr	Telephone interview
Italy-nr	Internet invitation
Luxembourg-nr	Telephone interview
Netherlands-nr	Telephone interview

## Austria

The target population were patients visiting neurologists or general practitioners (GPs) for any reason. Each of 200 members of the Austrian Neurological Society (ÖGN) and 400 GPs in Upper and Lower Austria and parts of Salzburg received ten questionnaires and were asked to distribute them to consecutive patients aged 18–65 years on one particular day of a pre-specified week. Whilst the sample was potentially 6,000, it was in fact probably smaller, since it was not known whether all questionnaires were actually handed out. The questionnaires distinguished between respondents recruited by GPs or neurologists. Patients were requested to complete and return them within 1 month; one reminder letter was sent to those who failed to do so.

## France

Here the project was performed with the cooperation of 80 GPs in the Voironnais region, which includes both urban and rural areas. Each GP received 30 questionnaires and distributed them to consecutive patients aged 18–65 years on one particular day of a pre-specified week. The questionnaires were completed in the waiting room and handed back in sealed envelopes. When this was not done, one reminder letter was sent by e-mail after 1 week.

## Germany

A list identifying a randomly selected population-based sample (*n* = 3,000) aged 18–65 years was obtained from the local municipal authority. For an urban/rural mix, half were drawn from the 580,000 inhabitants of the city of Essen in North-Rhine Westphalia and half from the 50,000 people living in the town of Kleve and surrounding villages in the western part of Germany. Questionnaires were distributed by regular post, with requests to complete and return them in postage-paid envelopes. No reminders were sent.

## Ireland

Questionnaires were sent by post by the Migraine Association of Ireland (MAI) to their 1,500 patient members; each was accompanied by a second questionnaire, distinguished from the first, to be completed by a partner or other non-biological relative. Reminders were sent electronically, and information about the survey was included in MAI’s newsletter and on their website. Recipients were asked to return the completed questionnaires in postage-paid envelopes. Each returned questionnaire was checked to ensure its appropriate origin (member or relative).

## Italy

The target population were the 330,000 inhabitants of Pavia province in the Lombardy region of Northern Italy. A stratified sample (*n* = 3,500), representative with regard to gender (1:1, F:M ratio), age (within the range 18–65 years), education and habitation (70% urban, 30% rural) was randomly selected in cooperation with Azienda Sanitaria Locale (ASL) of Pavia, the local health agency, who provided the sample list. Questionnaires were distributed by post, with requests to complete and return them in postage-paid envelopes. No reminders were sent.

## Lithuania

The target populations were the 352,000 inhabitants of Kaunas city and 89,000 of Kaunas region. A sample (*n* = 1,137) representative of the general population for age (within the range 18–65 years) and habitation (67% urban, 33% rural) was drawn by computer, using a stratified random sampling method, by the Residents’ Register Service. Data-collection by cold-calling (visiting households unannounced) was performed by medical students trained for the purpose, who personally interviewed consenting individuals following the structured questionnaire.

## Luxembourg

This country has 235,600 inhabitants aged 18–65 years. A sample (*n* = 6,498), representative of the population with regard to age (in this range), gender, nationality and habitation, was drawn by computer, using a stratified random sampling method, from the obligatory national social security registry of the Institut Général de la Sécurité Sociale. Questionnaires were sent by post in the language of the recipient (English, French, German or Portuguese), with requests to complete and return them in postage-paid envelopes. One reminder was sent to non-responders 1 month later.

## Netherlands

In the Netherlands, there were two surveys of two different target populations.

One (“Netherlands-population”) was executed by TNS-NIPO, a leading market research company with established access to a large sample of the Dutch population, representative with regard to gender, age, habitation, education and social status according to the standards of the Dutch National Bureau of Statistics (CBS). Questionnaires were sent through the internet to those aged 18–65 years within this sample (*n* = 200,000). Returned questionnaires with incomplete answers (other than those relating to income and body mass index) were automatically rejected. The study was stopped after 4 days, by which time sufficient questionnaires had been returned (from 1.2% of those to whom it was sent).

**Fig. 1:**
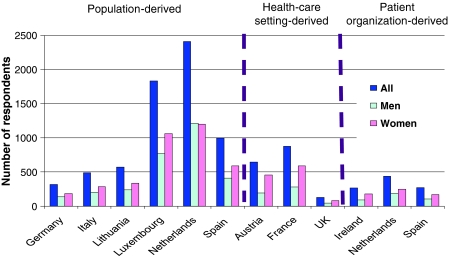
Participants per country in the different sampling methods of the survey

The second study (“Netherlands-patient”) targeted the 6,000 members of Nederlandse Vereniging van Hoofdpijnpatiënten (NVvHP), the Dutch headache patients’ organization. A computer-selected random sample (*n* = 500) was drawn from members aged 18–65 years in a male-to-female ratio of 1:3, excluding those with facial pain rather than headache. Questionnaires were distributed by post, accompanied by second distinguishable copies for household partners in cases where they were not themselves affected by headache; these would be a control group for future analysis. One reminder letter was sent after 2 weeks (Fig. 1).

## Spain

In Spain two surveys were conducted in different populations.

The first (“Spain-workplace”) was performed among a sample (*n* = 1,700) of employees of various companies operating in the tertiary sector (specifically the national postal services) living in ten areas of Spain: Albacete, Barcelona, Cadiz, Castellón, Cuenca, Ibiza, Palma de Mallorca, Teruel, Valencia and Zaragoza. The sample was stratified with regard to gender (male-to-female ratio of 1:1), age (within the range 18–65 years) and education. Ten occupational health physicians delivered and took return of the questionnaires. One reminder by telephone was issued to non-responders.

The second study (“Spain-patient”) was performed by the Asociación Española Pacientes con Cefalea (AEPAC), the Spanish headache patients’ organization. Helpers of AEPAC distributed 300 questionnaires personally among its members, and their families, living in and around Valencia. One reminder, by telephone or face-to-face, was given to non-responders.

## UK

The targeted population in the UK were patients of 12 general practices in 11 towns or cities: Aberdeen, Brighton, Cambridge, Cuddlington, Eastbourne, Exeter, Grantham, Guildford, Norwich, Tenterden and Weymouth. Each practice received 60 questionnaires to hand to consecutive patients attending for any reason. Recipients were asked to complete them in the waiting room and return them immediately, but they could also complete them later and return them by post. There were no reminders.

## Non-responder studies

It was likely, especially where responder-rates were low, that questionnaires would be returned preferentially by people most affected by headache, a potential source of significant bias. To estimate the probability and magnitude of this bias, 10% of all non-responders in Luxembourg, Germany and Italy (stratified for age, gender and, in Luxembourg only, nationality) and 10% of those in the Netherlands population-based study who replied within the first 4 days that they did not wish to complete the original questionnaire were re-contacted either by telephone or (in the case of Italy) by internet (Table 1, lower part). They were asked a few questions only: whether headache had occurred during the last year, three questions from ID migraine (a migraine screening instrument [13] allowing the identification of likely migraineurs) and questions on headache frequency and on headache, if any, on the previous day.

## Data entry

Independent double data-entry was performed in all cases: by two students in Austria, by two secretaries in France, by two students supervised by a physician in Germany, by a professional information officer and administrative staff in Ireland, in part by two members of the research team and in part by data-management personnel of CRP-Santé for Italy, by two members of the research team in Lithuania, by two students supervised by the scientific data-management leader of CRP-Santé in Luxembourg, by personnel from Het Ondersteuningsburo (HOB), an administrative support organization employed by NVvHP, in the Netherlands, by administrative staff of AEPAC in Spain and in part by two administrative staff members of Migraine Action UK and in part by data-management personnel of CRP-Santé for the UK.

## Data management and quality control

These were the responsibility of CRP-Santé, who issued detailed instructions on data entry and developed a database and means of electronic transfer via a secure web application to the central collecting point. All hard-copy completed questionnaires were also sent to CRP-Santé. These procedures were approved by the National Data Protection Committee in Luxembourg and tested for reliability and practicability during validation of the questionnaire [5].

The two sets of entered data from each country were compared at CRP-Santé and the discrepancies resolved by reference to the original questionnaires. The database was then locked prior to analysis.

## Results

The detailed results will be presented in future publications (along with analytical methods). Here we recount the results that reflect upon the methodology. Altogether, 9,269 correctly completed returned questionnaires were analyzed. They had a moderate female bias (58%: Table 2); of the respondents from the three patients’ organizations (*n* = 992), 61% were female. The mean age of all respondents was 44 years; samples from patients’ organizations were slightly older (mean 47 years) (Table 2).

**Table 2:** Responder-rates, gender distribution and mean age of samples in each survey

Survey	Denominator (*n*)	Responders (*n*)	Responder-rate (%)	Gender (% female)	Age (year) mean (SD)
Austria	Unknown, but not >6,000	646	Incalculable	70	48.8 (16.0)
France	2,400	876	36.5	68	50.2 (16.7)
Germany	3,000	338	11.3	57	44.6 (12.5)
Ireland	Members 1,500	195	13.0	66	49.4 (14.0)
	Relatives unknown	73	Incalculable		
Italy	3,500	500	14.3	58	43.4 (12.6)
Lithuania	1,137	616	54.2	59	40.9 (13.8)
Luxembourg	6,498	2,023	31.1	58	40.5 (12.7)
Netherlands-population	Unknown	2,414	Incalculable	50	42.6 (13.2)
Netherlands-patient	Members 500	337	67.4	57	48.6 (10.6)
	Partners unknown	115	Incalculable		
Spain-workplace	1,700	999	58.8	59	42.7 (11.9)
Spain-patient	300	272	90.7	62	41.6 (11.4)
UK	720	128	17.8*	65	48.0 (18.3)
Non-responder studies					
Germany-nr		260		55	Unknown
Italy-nr		202		70	39.4
Luxembourg-nr		357		50	Unknown
Netherlands-nr		188		52	38.9

The different sampling methods adopted in these ten countries worked with differing degrees of effectiveness, as evidenced by the responder-rates, which varied from 11.3 to 90.7% (Table 2). In the more population-based surveys, responder-rates varied from 11.3 to 54.2% (achieved in the door-to-door survey in Lithuania) and 58.8% (in the work-force population in Spain).

The samples were mostly of employed people of normal working age, and most were married or living with household partners.

Altogether, 1,007 people (51% female) participated in the non-responder studies. The responder-rates in these studies were generally high (Germany 80%; Luxembourg 87%; Netherlands 72%), although in Italy the denominator and, therefore, the responder-rate were unknowable.

## Discussion

An important feature of this study was that the same questionnaire was used in ten countries, constructed specifically for the project, revised after pilot studies, validated [4, 5] and translated into all local languages according to a rigorous translation protocol [8]. Although the diagnostic ability of the questionnaire was not assessed for accuracy within the project, the diagnostic question set had been used by LTB, in the local languages, for epidemiological studies in India (unpublished), China [8] and Russia [9]. In these countries, sensitivity and specificity were 63–77% and 82–99%, respectively, for migraine and 51–64% and 81–99% for TTH. Low sensitivity for TTH relative to diagnosis by headache experts reflects the fact that TTH is commonly infrequent (occurring less than once per month) and, therefore, not reported. Missing these cases makes little difference to estimates of impact.

To this extent the methods were constant. However, different sampling methods developed for the various surveys in these ten countries were the result of necessary compromises.

Truly population-based studies, ideally conducted door-to-door as in Lithuania, are highly resource-consuming and may be unjustifiable (even if practical) in a study of this scope and scale. There has to be regard for cost/benefit when considering levels of resource-investment, and this is what drove the compromises. In fact, the different methodologies were both a strength and a potential weakness of the study. They were a strength because different methods have different drawbacks, so the use of a variety of methods can yield more robust results overall. It would be a definite weakness if the purpose had been to compare the different countries, but this was not the case: Eurolight’s endeavour was aimed at estimating the impact of headache in Europe and, from that point of view, the mix of methods was not undesirable.

Less satisfactory were the variable and generally rather low responder-rates (Table 2), despite that the total sample size was large (*n* = 9,532). Low responder-rates may introduce bias. The key issue is whether samples were representative not only of the populations from which they were immediately derived but, more broadly and more importantly, from the general populations of the countries and of Europe (at least within the adult age range, usually 18–65 years, to which we restricted the surveys).

The highest responder-rates in non-patient groups were in Lithuania (54.2%), a door-to-door survey conducted by trained interviewers which, probably, provided more incentive to answer than the self-administration demanded elsewhere, and in the relatively captive work-force population of Spain-workplace (58.8%). We regard only the Lithuania study as truly population-based: all others were approximations, at varying distances, either because the sampling base was restricted as in Spain-workplace (a working population cannot be regarded as entirely representative of the full population even when the latter is limited to the working age-range) or because low responder-rates raised uncertainties about representativeness despite a broad sampling base (e.g., the 11.3% in Germany). In Italy and Luxembourg, samples were stratified for gender, age and habitation to achieve representativeness, but low responder-rates (14.3 and 31.1%) undermined this. In Netherlands-population, the sample contacted through the internet was representative but the study was stopped when only 1.2% had answered; the denominator (the number who had an opportunity to respond within the 4 days) was unknowable and, therefore, so are the responder-rates and degree of representativeness. In Austria, France and UK, samples were taken from patient populations, but not exclusively from headache patients. In UK, virtually all inhabitants are registered with their local GPs, so GPs’ lists are generally representative of the local population. Nevertheless, people with cause to visit doctors are, presumably, less healthy than the general population. They are also likely to be older, and this was borne out, and women were more highly represented (Table 2). Clearly the samples from members of patient organizations were not expected to be representative. What becomes of interest in these circumstances is how findings differed between the samples, even though this was not a purpose of the project.

Age and gender are important factors influencing the prevalence of headache, but imbalances in these are readily recognized and easily adjusted for during analyses. More problematic, because it is not only likely but also much more difficult to detect, is “interest-bias”. People with headache are more inclined to respond to a questionnaire about headache, and this inclination is, almost certainly, positively correlated with level of burden attributable to headache. This was the reason for conducting the non-responder studies: to detect at least whether the prevalence of headache was markedly lower amongst non-responders and, if so, to provide data whereby we could estimate uncertainties in the main findings.

The project was one of few so far to assess the separate impacts of migraine, TTH and MOH. It will, therefore, be possible to make comparisons between these. This may be of considerable interest, particularly with regard to time and productivity losses, effects on quality of life and financial costs. The samples were mostly of employed people, married or living with household partners. Good opportunities were created, therefore, to assess impact of headache beyond its effects on people with it: on work and productivity, and on family.

## Conclusion

The Eurolight project was the first at European Union level to assess the impact of headache disorders, and also the first project of its scale performed by a collaboration between professional and lay organizations and individuals. The methodology, although with differences born of necessity in the various surveys in the ten countries, was sound overall; biases should be detectable and their effects mitigated. In conclusion, we believe Eurolight will provide robust data revealing the amount of public ill-health that results from headache in Europe and carrying a very important message to health policy-makers.

## Appendix

Project Steering Committee: Colette Andrée (CRP-Santé, Luxembourg) (chairman and project leader), Zaza Katsarava (University of Essen, Germany), Miguel Lainez, (Fundacion para la Investigacion Biomédica, la Docencia y la Cooperacion Internacional, Valencia, Spain), Christian Lampl (Konventhospital Barmherzige Brüder, Linz, Austria), Michel Lanteri-Minet (CHU-Hôpital Pasteur, Département d’Evaluation et de Traitement de la Douleur, Nice, France), Elena Ruiz de la Torre, (Asociacion Española de Pacientes con Cefalea, Valencia, Spain), Timothy Steiner (Norwegian University of Science and Technology, Trondheim, Norway, and Imperial College London, London, UK), Lars Jacob Stovner (Norwegian University of Science and Technology and St Olavs Hospital, Trondheim, Norway), Cristina Tassorelli (Centro Italiano di Ricerche Neurologiche Applicate (CIRNA) and Headache Science Centre, National Neurological Research Institute C Mondino Foundation and University of Pavia, Pavia, Italy), Peter Vriezen (Nederlandse Vereniging van Hoofdpijnpatienten, Amersfoort, the Nederlands).

External Evaluation Board: Fabio Antonaci (President, EHF), Hilkka Kettinen (General Secretary, EHA), Peter Sàndor (Universitätsspital Zürich Neurologische Klinik, Zürich Switzerland), Jean Schoenen (Headache Research Unit, University Department of Neurology, Citadelle Hospital, Liège, Belgium).

Associated Partners: Gérard Mick (Réseau Voironnais de la Douleur), Daiva Rastenyte (Lithuanian University of Health Sciences, Kaunas, Lithuania), Anne MacGregor (The City of London Migraine Clinic, London, UK), Asociacion Española de Pacientes con Cefalea (Spain), Migraine Action (UK), Migraine Association of Ireland (Ireland), Nederlandse Vereniging van Hoofdpijnpatienten (The Netherlands), European Headache Alliance, European Headache Federation, *Lifting The Burden*: the Global Campaign against Headache.

Collaborating Partners: Judit Afra (National Institute of Neurosurgery, Budapest, Hungary), Marta Allena (National Neurological Research Institute C Mondino Foundation, Pavia, Italy), Bruno Carugno, (Azienda Sanitaria Locale of Pavia, Italy), Jean-Marie Gérard (Ligue Belge Contre les Cephalées, Nimy, Belgium), Heidy Hauser (Migraine Action Switzerland, Bottmingen, Switzerland), Carlo Lucchina (Regione Lombardia—Sanità, Italy), Giuseppe Nappi (National Neurological Research Institute C Mondino Foundation, Pavia, Italy), Konventhospital Barmherzige Brüder (Linz, Austria), Neurology Group of the Spanish Society of Occupational Practitioners (AEMMT), Austrian Neurological Society (ÖGN), Finnish Migraine Association.

## Abbreviations

EC: European Commission
EEB: External Evaluation Board
EHA: European Headache Alliance
EHF: European Headache Federation
EU: European Union
GP: General practitioner
ICHD-II: International Classification of Headache Disorders, 2nd edition
IHS: International Headache Society
LTB: Lifting The Burden
MOH: Medication-overuse headache
NGO: Non-governmental organization
PSC: Project Steering Committee
TTH: Tension-type headache
WHA: World Headache Alliance
WHO: World Health Organization
